# Principles of Military Surgery, Comprising Observations on the Arrangement, Police, and Practice of Hospitals, and on the History, Treatment, and Anomalies of Variola and Syphilis; Illustrated with Cases and Dissections

**Published:** 1830

**Authors:** 


*Principles of Military Surgery; comprising Observations on the arrangement, police and practice of Hospitals, and on the History, Treatment, and Anomalies of Variola and Syphilis. Illustrated with cases and dissections.* By John Hennen, M. D. F. R. S. E. Inspector of Military Hospitals. First American from the third London edition. With the life of the author, by his son, Dr. John Hennen. Philadelphia: Carey & Lea, 1830. p. p. 452.—8 vo.

It is with great pleasure that we congratulate the Medical Profession of our country on the appearance of this valuable work from the American press. Since the publication of the first edition, which came out in 1817, copies that have found their way across the atlantic, have been eagerly seized upon by surgeons, and have been treasured up as jewels of difficult acquisition—on three different occasions have we yielded to the instances of young professional friends in parting with our copy of the work, and on each occasion have we been obliged to wait a fresh importation for another copy.

Not only to the army surgeon is this a valuable treatise. By surgeons in practice in large cities—by those engaged in hospital service—but above all by the general practitioner of our Western States, Dr. Hennen's work needs but to be known, and it will be prized above all price.

Dr. Hennen was one of the first who detailed in a formal manner the results of that experience in surgery, which had accrued from those wars which, for nearly half a century, had devastated Europe. So early indeed as 1814, were we favoured by Professor Hall, of the University of Maryland, with a neat translation of the Memoirs of Military Surgery of the Baron Larrey, and thus was the professional appetite whetted for a continued detail of the wonderful results which a scientific examination of the ravages of war on the human frame had brought about. Before this, Mr. Cheraliers' beautiful little prize volume on Gunshot Wounds, was one of the earliest works on the military surgery of the period, after the immortal work of John Hunter. Mr. Guthrie, too, in 1814, wrote his valuable work on "Gunshot wounds requiring amputation," and the learned and indefatigable Professor Thompson, of Edinburgh, furnished us with his "Report on the state of the wounded in Belgium after the battle of Waterloo." All these were calculated to meet the wishes of the surgical practitioner, but there was still a desideratum—a regularly detailed treatise in which the various branches of military surgery should be presented, was most ardently desired—that the surgeon of other lands might learn what progress had been made in rendering the aids of science more applicable to human misery, and that the greatest possible benefit might result to suffering humanity, from the fierce and bloody struggle in which Europe had for so long a period been involved.

At this precise juncture the work of Mr. Hennen appeared, and was hailed with acclamations! No one perhaps was better calculated to fulfil this arduous task. A man of a clear and discriminating mind, elevated by force of native intellect above mean and despicable prejudice, thoroughly grounded in the elements of science, and above all, excited by an enthusiastic zeal for the progress and elevation of his profession. With such qualifications, and with the experience of twenty years in the actual practice of military surgery, which a high rank in the surgical staff of the army afforded him, and assisted by the contributions of his associates, he compiled the work which we are about to review.

It would be impossible to give any thing like a general view of Dr. Hennen's book in the limits usually assigned in medical journals to attempts of this nature. To present such portions of the work to the attention of our western brethren as will exhibit to them its value, is all that we design.

The work has gone through three editions in Europe. This, the last, from which the American reprint is taken, is published by his son. A short time before Dr. Hennen fell a victim to his exertions in an epidemic fever at Gibraltar, in November, 1828, he had prepared the materials for the third edition. These his son arranged for the press, wisely omitting the plates which had accompanied the preceding editions, and adding a biographical sketch of his learned and accomplished father.

The opening of the work presents us with a history of military surgery, or more properly with a chronological detail of the authors who have treated on this subject, remarkable as much for the purity and simplicity of the language in which it is clothed, as for the immense labour of research it developes.

The first chapter is devoted to the enumeration of the "*preparatory measures in taking the field.*" This, which it is not our intention minutely to examine, contains precepts, which, to the army surgeon about to enter into actual service, are of indispensable importance. His remarks on various instruments are highly instructive, and we feel assured that no surgeon will find himself the less wise for a careful perusal of them.

The second chapter opens with a detail of the various missiles employed in warfare, which is calculated to throw much interesting light on the subject of gunshot injuries.

* * * "In sieges, balls of very heavy metal (32 lb.) are employed; in engagements in open field, the largest shot seldom exceed 12 lb., and go down to 1 lb.; the other species of shot are the common musked ball, fired singly from muskets, or discharged in cases from field pieces; grape, which consists of small iron balls, disposed in linen bags fastened to a wooden bottom, in the middle of which is a spindle, round which the balls are secured by cord or wire; or case-shot, which consists of the same small iron balls put into tin cylinders, the bases of which are closed by two circular pieces of wood. The last kind of projectiles are shells, or hollow iron spheres, filled with powder, which may act either before or after their explosion.—The closer the contending parties are to each other, the more deadly will be the effects of all these balls; thus, according to D'Antoni, a 32 lb. shot may pierce a file of 70 men, a 16 lb., a file of 48, an 8 lb, a file of forty; a 13 oz. shot, a file of 20; a 6 oz., a file of 16; a 1 oz., a file of 4, if very close to them, and propelled by a certain degree of force,—while a shell will pass through from two to five men, and will kill or wound by its splinters, from six to nine; the distance and the resistance will of course produce great variation in the action of all these missiles. It must be confessed, however, that, much as the artillerist and engineer are interested in ascertaining these points with correctness, it leads to little improvement in surgery, except in as far as it shows the enormous violence with which the bones may be fractured, and their fragments dispersed either into the medullary cavities or the surrounding soft parts." p. 47.

There is so much of truth and simplicity in the following description of a gunshot wound, that no one can fail to recognize its accuracy:

"The effects of a gunshot wound differ so materially in different men and the appearances are so various, according to the nature of the part wounded, and the greater or lesser force with which it has been struck, that no invariable train of symptoms can be laid down as its *necessary* concomitants. If a musket or pistol ball has struck a fleshy part, without injuring any material blood-vessel, we see a hole about the size of, or smaller, than the bullet itself, with a more or less discoloured lip forced inwards; and, if it has passed through the parts, we find an everted edge, and a more ragged and larger orifice at the point of its exit; the hæmorrhage is in this case very slight, and the pain inconsiderable, insomuch that in many instances the wounded man is not aware of his having received any injury. If, however, the ball has torn a large vessel, or nerve, the hæmorrhage will generally be profuse, or the pain of the wound severe, and the power of the part lost. Some men will have a limb carried off or shattered to pieces by a cannon ball, without exhibiting the slightest symptoms of mental or corporeal agitation; nay, even without being conscious of the occurrence; and when they are, they will coolly argue on the probable result of the injury: while a deadly paleness, instant vomiting, profuse perspiration, and universal tremor, will seize another on the receipt of a slight flesh wound. This tremor, which has been so much talked of, and which, to an experienced eye, is really terrifying, is soon relieved by a mouthful of wine or spirits: or by an opiate; but above all, by the tenderness and sympathising manner of the surgeon, and his assurances of his patient's safety. Where some important or vital organ is injured, considerable pain and much anxiety is a general consequence; these will be more particularly considered in treating of the wounds of particular parts.

If the ball has passed through the fleshy part of the arm, thigh, or buttock, we do no more than sponge the part clean, place a small bit of folded lint on each orifice, which we retain by two cross slips of adhesive plaster, and lay over, at most, two or three turns of a roller. The ball will frequently have passed nearly through the limb, and be retained only by the elasticity of the common integuments; there we cut upon and extract it at once; and we should lay it down as a rule not to be deviated from, to extract on the spot every extraneous body that we possibly can, either by the forceps alone, or with the aid of a bistoury. But those who best know the field of battle will easiest admit how often it is impossible to do all in this respect that they could wish." p. 48—49.

The various courses which balls will mark out is very curious. A musket ball will strike a man on the front of his cap, pass round and carry away the hinder tassel. Dr. Hennen is the first, we believe, who noticed that balls will course along *concave* as well as *convex* surfaces; as for instance between the pleura costalis and the lungs. A very slight resistance will cause a ball to turn from its course. We remember a sergeant of artillery who had a most protuberent abdomen, who received a ball in that part, which appeared under the skin on the other side, and which was supposed to have passed through the cavity. The ball was cut out, and the red line which the next day showed itself between the two openings, plainly indicated that the ball had passed between the skin and the fascia superficialis, without penetrating the belly.

We ought to bear in mind the vast difference between the effect produced by a musket and a rifle ball. As the rifle is peculiarly an American instrument, and one too, which, in the hands of our western hunters, is unerring in its effects, a moment or two devoted to the motion of the ball and the effect it produces, will not be time misspent. The motion of a musket ball, independently of its projectile course, on its own axis, is at right angles with its direction. A very slight knowledge of the laws of motion explains this phenomenon. The consequence of this motion is, that when a musket ball strikes the flesh, the hole made is smaller to all appearance than the ball itself. The barrel of the rifle is grooved not in a longitudinal direction as the French and German rifles, but in a spiral manner. The ball is forced down so tightly, that as it passes out, it is under the necessity of following the course of the spiral groove. This imparts to it a motion on its own axis, corresponding with the direction of its course. Besides, the whole ball follows a spiral direction, forming in its progress a hollow cylinder, if I may be permitted the expression. Hence the *ragged hole*, which our hunters know so well, is always much larger than the ball. Hence the rifle ball at full momentum, does not, like the musket ball, remove a cylinder of muscle and bone, but by its rotatory motion tears the flesh and shatters the bone. Hence too, unless the ball is nearly spent, it never glances. These peculiarities in the effect of a rifle ball, are highly interesting to us as Americans. We regret that the limits of this paper, and the importance of other matter before us, do not allow an extension of these remarks.

Before leaving the subject of the glancing of balls, we take occasion to mention a singular instance of mistake in a military surgeon. An officer of distinguished bravery, during the late war, led the forlorn hope of an army into a wood that was supposed to be filled with Indians. His party had scarcely entered the wood, when a well directed fire from the hidden enemy, unhorsed every man, except the gallant officer, who was wounded by five balls. When he was removed from the field, a ball was found to have entered his hip, and another passing through the saddle had touched the tip of the glans penis. Now the sapient surgeon supposed that the ball which had entered the hip, had found its way out through the urethra!!

Mr. Hennen next, in a most forcible manner, proceeds to illustrate and explain the necessity of primary amputations; that is, amputations on the field of battle. It would not be doing justice to pass over the portion of military surgery without a commendation of the chivalrous enthusiasm and fearless bravery of the French surgeons who uniformly established their field hospitals in the rear of the army, and who flew to the assistance of the wounded under showers of grape shot, in the face of the most appalling dangers. It is singular how successfully that transcendant genius, Napoleon, succeeded in infusing into his men and his officers a high minded recklessness of danger when in the discharge of duty.

The remainder of the chapter is devoted to the consideration of sabre cuts, contusions, &c.

*The preparatory arrangement and selection of the fixed and receiving Hospitals* is the subject of the 3d chapter. We would strongly recommend this portion of the work, which is replete with information of the most valuable nature to those who may devote their time and medical attainments to the service of their country.

Dr. Hennen in his 4th chapter speaks of the *dressings, and general medical treatment.*

"There is no urgent necessity for removing the dressings which have been applied in the field to the more simple wounds of the extremeties, for the first two or three days, whether the wounded have arrived in the hospital, or are only on their passage to it, provided the slips of plaster and bandage are sufficiently secure, the dressings unstained by a sordid bloody oozing, or no serious stiffness or uneasiness is perceived in the part by the patient himself. In this, however, we must be guided by season, climate, and the constitution of the individual, or the peculiarity of his wound. It will generally be sufficient to keep the dressings moistened with cold water, either alone, or mixed with a little spirits, vinegar, or wine; or, if the weather demands, and convenience on the march permits, the same moderately warmed. As soon as possible after this period, the field dressings should be removed, and the limb either covered with cloths, moistened with an appropriate liquid, or laid in emollient poultices moderately warm. It has of late years become a fashion to decry the application of poultices, and to dwell on the harm they *may* produce, putting entirely out of view the essential service that we actually derive from them; but after long experience on this point, and judging from the feelings of the patients themselves, and the obvious effects upon their wounds, I have no hesitation in saying, that a soft and moderately warm poultice of bread, meal, bran, pumpkin, carrot, or any other emollient substance, carefully applied, and removed at least twice a day, until the sloughs begin to loosen at the edges, and purulent oozing is seen issuing from under them—in fine, till the process of suppuration is fairly commenced, is the best and most appropriate remedy in the early stages of simple gunshot wounds, attended with much contusion of the soft parts, and high inflammation. They should not be continued after this period, nor should they at any time be applied, except under the direction of the attending surgeon. It is to the abuse of continuing poultices day after day, indiscriminately to all states and stages of wounds, that their rejection is to be attributed, and that their bad effects are due. If the inflammatory symptoms do not run very high, and that the sloughs are beginning to separate kindly, a pledget spread with any simple ointment, or merely dipped in oil, and covered with cloths moistened in acidulated water, will be quite sufficient as an external application; while the general state of the system should be cautiously attended to in all cases. Compresses dipped in simple cold water have lately been much recommended by Kern and Assalini, as a substitute for almost all other dressings; and I have seen them employed with considerable advantage. Where this plan is adopted, oil-skins should be employed at the same time, otherwise the beds get saturated with moisture, and severe pulmonary attacks, or rheumatism, may ensue." p. 71—72.

The doctor enters into the history of the use of cold water, in dressing gunshot and other wounds. It is not our intention to touch this subject further than to state that the Baron Percy, late chief surgeon of the armies of Napoleon, had the honour of reviving this antiquated practice with most astonishing success. In the article "Eau," in the Dictionaire des Sciences Medicales, the Baron has detailed the wonderful results of this simple practice.

The mode of conducting the subsequent dressings is next exhibited, and our author strongly recommends the admirable observations to be found in Professor Thompson's Lectures on Inflammation, relative to the management of dressings, a recommendation in which we most heartily unite.

The 5th chapter is devoted to the *extraction of foreign bodies.*—Into this subject we do not mean particularly to enter, farther than to state our belief that the common dressing forceps, and the simplification of Baron Percy's bullet forceps, as employed by the surgeons of the United States' troops, will be found fully adequate to all our wants, and far superior to more complicated and more costly instruments.

A very curious case we must be permitted to extract.

' "The particulars of the wound," says Dr. Kennedy, "by which Lieutenant F——, of his majesty's 12th regiment, was killed at the siege of Seringapatum, were stated to me a very few days afterwards, by the late Dr. Alexander Anderson, the superintending surgeon and chief medical officer of the army, as follows:—

"A shot from a heavy gun came rolling along the ground, like a spent ball, towards the trenches. It rolled over that part of the banquet under which Lieutenant F—— happened to be lying down, and buried itself under the skin and muscles of his hip. He was immediately put into a dooly and carried into Dr. Anderson's tent. Upon laying down the dooly, the bearers complained of the difficulty they had found in carrying it from the trenches, owing to its having been unusually heavy on one side. Dr. Anderson, upon running his fingers into the wound, was surprised to find a mass of iron of such unusual size, that he concluded it must be part of a large shell which was lodged there. Lieutenant F—— being then moribund, the shot was not cut out till after he died, when it proved to be what Dr. Anderson called to me unequivocally a *thirty two* pound shot. One circumstance only throws any doubt upon its having been actually a shot of this calibre, and it is this:—It was afterwards said that this shot had been fired from a gun very conspicuous, during the siege, both from its being mounted upon a high cavalier, and also from the mischief it did; and it was also said, that after the place was taken, this gun was found to be only a French twenty-four pounder, which gives a calibre of nearly twenty eight pounds English. Whether this shot which killed Lieutenant F—— was fired from this gun I do not know, but it is certain that a shot thrown from this very gun into the head quarter line, (which was an unusual distance,) and which lay during the rest of the siege near to Lord Harris's tent, was afterwards looked upon and spoken of as a thirty-two pound shot."' p. 82—83.

Chapter 6, *on Contusions and other serious injuries from shot and shell*, is a most interesting one. The subject of Contusions is one of the greatest importance, and one which has been too much neglected in the formal works on Surgery. It is an extensive and important field which as yet lies open, and we most ardently hope that some surgeon of great experience and research, will deem the subject worthy of a monograph.

The 7th chapter, on *injuries of the bones*. Amid the mass of useful information which this volume contains, it might be difficult to select a chapter to which the meed of superiority should be awarded. This one before us is perhaps *practically* the best. To publish the whole chapter would only be doing justice to its merits. We mean however to touch only a few points. Compound fractures, the result of gunshot injuries, are of course very complicated in their nature. A gunshot fracture of the thigh bone is so serious in its consequences, that it has been emphatically called *the compound fracture*. Mr. Guthrie, on reviewing the cases that occurred under his care in the British army while in the peninsula, found that not more than one sixth of the men whose thigh bones had suffered compound fracture, recovered so as to have useful limbs. Two thirds of the whole died, whether the limb had been amputated or not, and the remaining sixth dragged after them useless limbs which were constant sources of irritation.

Dr. Hennen's mode of treating compound fractures is founded on correct pathological views, and it is very important that it should be extensively known. The common practice in these cases is to reduce the displaced extremities of the bone, to swathe the limb up tightly with a many-tailed bandage, and perhaps to apply the instruments of permanent extension. This is done immediately after the accident, for in general, surgeons seem to have imbibed the vulgar prejudice that nothing is done till *the bone is set*. Now what will be the result of such a course?

The inflammatory symptoms which in this case will run very high, begin to come on. The pain increases, and the swelling too. The limb has been tightly bandaged up, and there is not room for the diastasis, and the compression produces the most violent agonies, while the surgeon stands by his tortured patient, pouring forth the consolations of philosophy, and delineating the beauties of resignation to the inevitable destiny. Well, the patient gets along to the third or fourth day, and the surgeon cautiously undoes his scultets, when lo! livid vesications are peeping between the folds of his bandage, and a large slough surrounds the lacerated wound. This is no exaggerated picture. But too many woful instances have come under our observation. Even where these horrible results do not follow, much misery and protracted suffering is brought about, by keeping the limb bandaged, and in a constrained posture during the inflammatory symptoms. Every tyro in surgery knows, that while inflammation is present in compound fracture, the formation of callus does not go on. During this violent reaction, the process of ossification is completely at a stand, and indeed during the whole of the cure, high inflammatory and suppurative action will prevent the formation of bone. This violent inflammatory stage lasts from four or five days to two weeks.—During this period, Dr. Hennen places his patient according to Mr. Pott's mode, in a relaxed position, removing the splinters of bone and other extraneous matters, applying a cloth wet with water, or with camphorated or saturnine lotion.

* * * "When the inflammatory symptoms are subdued, I then proceed more accurately to adjust the fractured bone; and to this end, place the patient on his back, a change of posture which invariably gives relief, filling up all hollows, and arranging the limb precisely after the mode recommended by Professor Boyer, and applying the bandage of Scultetus afresh, with a roller moderately tight, on the lower portion of the limb, and proper compresses along the parts. I employ the improved splints of Assalini, if they can possibly be procured; if not, I place two common long splints in the usual way—one from above the hip to the ankle, or from above the knee to the ankle, as the case may be, and the other of proportionate length on the inside; and, fixing the pelvis of the patient by a bandage to the upper part of the bed, (if overlapping of the bones renders such extension necessary,) I stretch out and retain the limb by means of tape fixed to the bottom, or what I have found answer still better, by a common tourniquet, the centre of its strap firmly fixed round the knee or ankle, and buckled over the bed post, so that, by turning the screw, the extension may be moderately made and increased as circumstances demand.—This, which was suggested to me by Professor Thomson, at Brussels, I have found of very great assistance in some obstinate and complicated cases. In this mixed mode we reap the advantages both of the position of Mr. Pott, and that of Pare and the more modern French surgeons. The patient is in general extremely tired of his relaxed position before the lowering of the inflammatory symptoms indicates the time for placing him on his back, a change from which he receives great relief; we may rest assured that the process of ossification has not commenced until that period; and that, consequently, the application of machinery to extend the limb, or splints and bandage to confine and regulate the new callus, is unnecessary, if not hurtful.

The speculative objection that may be offered against the plan of leaving the bones so long unset, is the possibility of an irregular or distorted union; but with daily attention, this can scarcely occur. I have only met with one instance of it; it was in an officer who would admit of no treatment but what he himself deemed proper. In consequence of this, he very nearly paid the forfeit of his life in the first instance, and he is now obliged to wear a high heeled boot, the thigh being considerably shortened and curved." p. 108—109.

For ten years we have been in the habit of treating compound fractures on Dr. H's principles, and the result of an extensive experience is most triumphantly in its favour. We have also extended the same principle to the treatment of simple fractures, and with equal success. We cannot dismiss the subject without again cautioning our readers against *tight* bandages in all cases of fracture. The tight bandage is a relic of the old barbarous modes of surgery; is founded on the irrational idea of moulding the callus, and is gradually sinking into disuse and oblivion.—Other modes of keeping the fractured ends of bones in contact, are now happily in general use. We must take our leave of this most instructive chapter, without having even glanced at one tenth part of its valuable matter.

*Injuries of the joints* are treated of in the 8th chapter, which is replete with curious and highly interesting matter.

The 9th chapter is devoted to the consideration *of contracted extremities.*

"To remedy these contractions, a variety of mechanical contrivances have been had recourse to; but I am persuaded, that the more simple the plan, provided it has sufficient power, the more likely it is to succeed. That in use at Hilsea hospital, is perhaps the most simple of any, and can be easily, and without expense, erected in every hospital. It consists of a firm wooden chair, with a strong jugum of wood, of the shape of the letter U, for confining the thigh, if the contraction is in the knee-joint; or a piece of wood with a hinge, like the letter V, if the elbow be the joint affected; some small brass pulleys made for screwing in the floor, wall, or ceiling, with a proper cord; a shoe with three loops of iron, one at the toe, and one at each side, half way between the toe and heel, and a tin or other vessel for containing some weights. The inventor of this very simple machine I do not know, but of its utility, under Staff-surgeon Coates, and Dr. Knox, I have had several most convincing proofs.

The mode of using it for a contracted knee, is as follows:—The patient being seated on the chair, the jugum, properly cushioned, is fixed over the thigh, so as to prevent it from rising, and the shoe being put on, and steadied by cords and hooks applied to the external iron loops, and fastened to the wall, a cord of whatever length may be judged proper, previously brought through one or more pullies, and, with a hook attached to the end of it, is fastened to the loop in the toe of the shoe; at its other end is the scale or vessel for holding the weight. Now, it is evident, that, on putting a weight into the vessel at one end of the cord, the other must be acted upon; and if the weights are properly graduated, force to any degree, from the most gentle, can be made use of. In the same way, the arm being secured in the hinged jugum, by straps to a proper table, the cord hooked in the jugum, and first passed through a pulley in the floor, and then through one in the ceiling, as in the other case, will have the effect of gradually stretching it. The advantage gained by each trial can be measured by a common ruler. Simple or medicated frictions, or the affusion of cold or tepid water, may be employed during the time of using the pulley, and the limb may be subjected to it as often during the day as may be thought necessary. Morning and evening are the usual periods at Hilsea, and hitherto there have been no states of contraction, except those depending on anchylosis, which have not derived benefit from it.

By a judicious application of bandages, and a strict attention to posture, in the early stages of wounds, these accidents may in most cases be prevented; and in all, their future ill effects considerably lessened; but it is a matter of serious importance, that a surgeon, unacquainted with military practice, should be put upon his guard against their voluntary occurrence, the frequency of which in the army has no parallel in the records of civil hospitals. The judgment and discretion of the surgeon will point out to him the means to be adopted in each individual case; it may not, however, be amiss to mention a few leading points. Permanent contractions in the joints of the fingers, and rigidity of the flexor tendons, will be always best guarded against by laying the hand flat out on a splint of wood adapted to its general shape, digitated at its extremities, and properly secured; while, in the intervals of the dressings, a gentle motion of the fingers, thumb, and wrist, should be encouraged in the patient, or if, as often happens, he obstinately objects to it, effected by the hand of the surgeon himself. This should be done with tenderness and caution, wherever the injury may be; but if the alleged contraction or rigidity is attributed to a wound, through a part where the principal nerves have not been injured, and the muscles of which have evidently no power over the joint affected, all tenderness is criminality." p. 141—142—143.

The tenth chapter treats of *injuries of the blood vessels.* The commonly received opinion is, that the hœmorrhage from a gunshot wound is but trifling, and that every bleeding gunshot wound is almost necessarily fatal. A ball opening a large artery will at times give rise to a dreadful bleeding; at the siege of Saragossa, Professor Assaline saw a surgeon expire in a few moments whose carotid was touched by a musket ball. That such is not always the case the following extract will serve to show.

'How the vessels escape so often as they do, has been a source of great surprise among surgeons, and is truly wonderful. Balls buff along them, pass between them, and traverse their courses in all possible directions, but leave them unhurt. Their elasticity has been considered as in a great measure contributing to this effect, and no doubt it does; something also may depend upon their being in a state of dilatation or otherwise when the missile passes across them, but still much is left unexplained in the attempt of accounting for these phenomena. Where a round shot injures a large vessel, or a sabre divides it, (the carotid for instance, or the femoral,) immediate death is almost universally the consequence. Where a vessel of this class is opened within the cavities, no chance of recovery remains; the cause and effect are almost simultaneous, and even in the first case death amounts so near to certainty, that a bare possibility of escape is left, and no practical deduction can be drawn from a few solitary instances to the contrary. It must be recollected, however, that if a limb is entirely carried off, or if it is excessively bruised, little or no bleeding takes place: the vessels are paralyzed, and their organization almost destroyed; the effect is nearly the same as if they had been actually seared, a process to which the older surgeons, who were so familiar with hot irons, often compared it. A very singular instance of escape is given by M. Larrey in his Memoires, in which an aide-de-camp received a gun-shot wound, which cut the external carotid at its separation from the internal, and at its passage through the parotid gland. The immediate application of pressure from the fingers of an intelligent soldier upon the spot, and M. Larrey's subsequent bandages, saved the patient. I know of an English officer who was also saved in India from the effects of an arrow wound in the carotid by the same means. In the 9th volume of the New Medical and Physical Journal, p. 95, is given the case of a man wounded in the femoral artery at Spithead, and saved by a subsequent operation at Haslar hospital ; and in the 3d volume of the Medico-Chirurgical Journal, p. 2, is given an instance of the carotid bursting and being taken up on the spot by the late Mr. Fleming, a naval surgeon.

We have occasional opportunities in the field of at once trying the extremities of lacerated arteries, and these should never be neglected, although, perhaps, in every case nor indispensable. These occur where a limb has been carried of by a cannon shot, and where the vessels hang out like cords from the wound, without the least flow of blood from them ; or where the extremities of the wounded vessels can be seen partially throwing out their blood in the bottom of a deep wound, In these, and in all other cases of divided arteries, the least reflection upon the anastomoses of the vessels will lead us to secure both the end next the source of circulation, and the more remote extremity. Mr. Guthrie, in his excellent cases inserted in a periodical publication, has called the attention of military surgeons to this point, and his observations cannot be too forcibly impressed on their minds. But many cases occur where nature has herself completed the entire process of cure without any interference of the surgeon. I owe the following to that very able and experienced officer, Dr. Dickson of Clifton, physician to the fleet:

'Sergeant Kelly, of the 54th regiment, in the battle of the 18th of March, 1801, in Egypt, had both arms shot away at the elbow joints by the same cannon ball, and on the following evening was received, with a great number of wounded men, on board the Braakel, of which ship I was surgeon. On removing the dressings, I was much surprised to find no appearance of a single blood vessel being tied by ligature. Conceiving, I imagine, the case to be desperate, a piece of linen had been merely wrapped round each stump, and the supervention of syncope seems to have saved his lifo, by stopping the farther effusion of blood. The left arm, which was the most ragged, and having the olecranon still attached, was amputated ; but from the low state to which the man was reduced, and the pressure of other cases, I was obliged to postpone doing any thing with the other arm; and, considering, after the time had elapsed, that there was but little danger of bleeding, and wishing to see the result, a tourniquet was merely put on loose. The second operation could not be performed until the 25th, and the dissection of the amputated portion then satisfactorily showed that ample provision had been made against the possibility of such an occurrence ; for the end of the torn artery was completely obliterated and lost in the surrounding flesh, and for upwards of an inch from its extremity was described, by the surgeons who assisted me, to be solid, organized, and carnous. I say described, for while on other duty, it had been thrown overboard. The poor fellow fevered, and died on the 4th of April.' " p. 146—148

The true practical value of the following quotation will come home to the experience of every surgeon who has seen much of wounded arteries.

"It is in the hospital that our most guarded attention is called for, and our most saving efforts made, in the expectation, or actual occurrence of secondary hemorrhage. In these cases, we must avail ourselves of every assistance which the history of surgery has from time to time presented to our notice. To pressure, to cold, and to styptics, we must give their due value, and a full trial to such an extent as prudence may warrant ; but it is to the actual ligature of the vessel that we are to trust in all cases where a trunk or important branch is injured ; and in cutting for them, correct anatomical knowlddge is our only resource. Often, unfortunately too often, we find, that even this will not enable us to meet the exigencies of actual practicc, and that the mere anatomist has made but one step to being a perfect surgeon; a principal one, indeed, but no more conclusive, than the tributary arts of the chisel or the pencil are to the formation of a perfect architect. Much unmerited blame has been thrown on the army surgeons, on this as well as other points, by men who, with a minute knowledge of the natural structure, have not adverted to the pathology of wounded vessels. They prick for arteries in a dead subject, and they readily find them ; but the state of a blood vessel in a wounded limb is very essentially different from what it is in a sound state, or in a body laid on the table for the practical purposes of anatomy ; and I have more than once seen a person of this class, after having cut upon a living blood vessel with the utmost precision, and described its course with the most laudable minuteness, confess, with great surprise, that he was unable to secure it, and had actually left his patient much worse than he found him. The state of general health, the cause, extent, and nature of the wound, the diseased state of the vessels, or their natural connexions, the "engorgement" of the parts with extravasated blood, or putrid sanies, the possibility of irretrievably injuring the limb by cutting its nerves or the tendon of some muscle essential to the due performance of its motions, should all be miuutely examined, and balanced in the operator's mind ; and he will have certainly the best claim to the character of a judicions surgeon, who saves the limb of his patient, if possible, but who does not hesitate between the probable salvation of it, and the certain loss of life." p. 149.

Dr. Hennen takes up the consideration of the modes of using the the various kinds of ligature. After the introduction of the ligature as a mode of arresting hemorrhage by Ambrose Parey, the next improvement in real utility was made by our friend Dr. Veitch, of London, who has attained so much celebrity by his highly valuable work on yellow fever, and whose labour in another surgical improvement will again come under our notice before we leave Dr. Hennen's book.

This consisted in cutting off one end of the ligature after it is tied ; a practice which is now very common. Dr. Hennen seems at a loss to know whence origiuated the practice of cutting off both ends of the ligature. He supposes it to have occurred accidentally to the mind of some American naval surgeon, who mentioned it to assistant staff surgeon Hume. The idea of employing an animal substance as a ligature first originated with our distinguished American Surgeon, Professor Physick, of the University of Pennsylvania. In 1806 he tied the femoral artery of a horse, cutting off both ends of the buckskin ligature. The wound healed rapidly, and nothing more was ever heard of the ligature. Since that period the Professor has used in all his numerous operations, ligatures made of fine white French kid, from which the grain is peeled off, cut into strips, soaked in water, and then rolled so as to become round and smooth. Since that period, (about 1813,) the short cut ligature was practised in England. It is much to be regretted that Dr. Physick, content with the universal estimation in whieh he is held, does not communicate more fully to his brethren, the result of his vast experience, and that he does not more manfully vindicate his claim to the many bold and original improvements he has introduced into surgery. Ligatures ofsilk, when cut short off at the knot are generally absorbed, yet we think the animal ligature is mostly preferable to the silken one.

Chapter XI. *Injuries of the Nerves.* We simply make an extract from this chapter, of a remarkable case in which nerves were included in the ligatures.

"A general officer, of distinguished gallantry, was struck by a round shot during a very desperately fought action, which buffing along his breast in an oblique direction, destroyed the arm, and left only the head of the bone and a very small portion of the shaft remaining. He was carried to an adjoining hovel, where the common amputation was performed under very unfavorable circumstances; the night was coming on, the supply of candles was scanty, and the enemy's shot were flying in all directions. The general was placed under my care on the day after the operation. The variety of cross accidents from fever and extensive sloughing, it is not within my purpose at present to enlarge upon, but the first attempt at clearing the ligatures, and making gentle pressure on them, was attended with pain so excruciating, as to leave no doubt that each included a nerve, or was in a certain degree connected with some large nervous filaments. This agonizing sensation was not felt except the ligatures were pulled at, and then not in the stump itself, but was referred to the finger, thumb, wrist, elbow, or even to the external skin of the lost arm, as one or other ligature might be handled. I have sometimes been led to think, that the general uniformly felt the same sensations when the same ligature was touched, as I generally made my attempts to extricate them in a regulated succession, and his complaints were often of the same succession of parts. More attentive observation, however, convinced me that this was not the case; for if any one was pulled with more steadiness than another, he complained of all the parts suffering pain simultaneously. One small ligature, if pulled in an oblique direction inwards, towards the axilla, always gave him imaginary pain about the elbow or in the skin; but if the same was pulled strongly and directly downwards, the fingers were complained of. He has, frequently after the smarting of dressing was over, with great accuracy pointed out on my arm the course of the internal cutaneous nerve, as the site of his ideal pain; often he has described that of the external; and, on one occasion, I with utter astonishment, had the general neurology of my arm and fingers traced by him. But unless the ligatures were pulled at, he had no other uneasy sensations than those which usually occur in persons whose limbs have been amputated. Once only did I ever know him to refer his pain to the seat of the sensorium itself. On that occasion, from using an artery forceps to the ligatures, on which the slide moved rather stiffly, I exerted a greater force than I had intended. He convulsively put his hand to his head, expressed a sense of exquisite pain in his brain, involuntary tears dropped from his eyes, a paralytic contraction momentarily affected his mouth, a universal paleness spread over the uncovered parts of his body; and although universally tolerant of pain, and of most remarkable equanimity of temper, he uttered a piecing cry, and exclaimed, "that the agony in his head and neck was insufferable." The state of collapse was so great, that I was obliged to send an aid-de-camp instantly for volatile alkali, and a glass of Madeira, by which he was soon relieved: but the painful sensation, and the prostration of his strength, continued through the day. A British admiral was present on this and various other occasions, and observed to me, after I had confessed my inability to explain, even to my own satisfaction, the cause of all these sensations, "that he never saw the general dressed without applying mentally to the wonderful sympathy manifested on those occasions, the expression of Pope : 'it lives along the line.' I believe we must be content with the fact, without seeking for the explanation.

To account for the mode of action of the nerves, or the connexion between mind and body, will perhaps never be permitted us in our present state of existence : and we, who, to use the language of the Swedish philosopher, 'have calculated the laws of motion for distant worlds,' are in profound darkness on important points connected with our own. There is no doubt, however, that the principle of perception exists in full integrity in the cerebral mass, even after these "internunciate chords" are divided. The investigation of this most curious subject belongs to the physiologist : unfortunately, the little purpose to which it has hitherto been pursued is but too obvious. It remains for me to say, that, after various gentle attempts at cutting, pulling, twisting, and a graduated and constant strain by means of appending small weights, or tying the separated threads of the ligatures over little quills of plaster, and similar contrivances, fully a year elapsed before the last ligature was removed by my friend, Dr. Irwin, deputy inspector of hospitals. The general's health did not suffer ; and the unanimous opinion of the best informed surgeons was, to try no violent measures or extensive incisions, but trust to constant gentle means and the slow operations of nature. An experiment of placing a ligature on the axillary plexus, or on any single nerve in the dead subject, will show what an obstinate resistance is offered after the protrusion of the medullary substance, by the subsequent puckering of the tough investing membrane, which during life will not admit of the ligature sliding off, either until the part is absorbed in course of time, or the materials of which the ligature is composed undergo some decomposition." p. 164—166.

Chapter XII contains notices *of some general affections of the system from wounds.* The symptomatic inflammatory fever is first treated of and then the hectic form of symptomatic fever. The Doctor next enters at large into the nuisances of hospitals and the preparatory steps on the entering of the sick and wounded. Great attention to cleanliness is requisite, and it is strongly enforced by our author. The practice in the Infirmary of the Philadelphia Alms House is highly commendable and worthy of imitation. On admission the patient has his hair cut, undergoes the lustration of the warm bath, with a plentiful accompaniment of soap and thorough wiping down with towels, is then clad in clothes perfectly clean and fresh. The violent aversion of the inmates of a hospital to fresh air and free ventilation, is a fertile source of many of the forms of disease to which such establishments are peculiarly liable. In speaking of the tenacity with which the effluvium of confined and crowded apartments adhere to the substances exposed to them, Dr. Hennen says :

"The late illustrious philanthropist, Howard, gives a most striking proof of this, in his observations on the air of prisons. 'My reader,' says he, 'will judge of its malignity, when I assure him, that my clothes were, in my first journeys, so offensive, that in a post chaise I could not bear the windows drawn up ; and was, therefore, obliged to travel on horseback. The leaves of my memorandum book were often so tainted, that I could not use it till after spreading it an hour or two before the fire : and even my antidote, a vial of vinegar, has, after using it in a few prisons, become intolerably disagreeable.' —'Dr. Hales,' he adds in a note, 'Sir John Pringle, and others, have observed, that air, corrupted and putrified, is of such a subtle and powerful nature, as to rot and dissolve heart of oak ; and that the walls of buildings have been impregnated with the poisonous matter for years together.' Some of the villages in Portugal, which had been occupied as hospitals during the peninsular campaigns, became so saturated with contagion, that a few hours residence insured to many a paroxysm of headache or fever, if a copious bilious vomiting or diarrhea did not prevent its accession." p. 173.

Fumigation next comes under consideration. This subject we do not mean to touch.

A short extract which Dr. H. introduces from Humboldt's personal narrative shews the value of free air to the sick in confined apartments.

"A sailor," says he, "who was near expiring, recovered his health from a circumstance that is worthy of being mentioned ; his hammock was so slung, that there was not ten inches between his face and the deck. It was impossible to administer the sacrament in this situation, for, agreeable to the custom on board of Spanish vessels, the viaticum ought to be carried by the light of tapers, and followed by the whole crew. The patient was removed into an airy place, near the hatchway, where a small square birth had been formed with sail cloth ; here he was to remain till he died, which was an event expected every moment ; but passing from an air extremely heated, stagnant, and filled with miasma, into fresher and purer air, which was renewed every instant, he gradually revived from his lethargic state, and his recovery dated from the day when he quitted the middle deck." p. 175—176.

The Doctor's own case is so interesting that we cannot pass it by.

"I may perhaps, be permitted to adduce my own case, in further illustration of a subject, which can never be enough impressed on the army surgeon. While the British army was encamped upon the heights of Sobral, covering the approach to Lisbon, and watching the movements of the French under Marshal Masena, in 1810, it became a matter of necessity to have the whole in a state of preparation for movement at the shortest notice. Our baggage, therefore, was always ready packed at night, and we remained ready to turn out at a moment's warning. I procured what I conceived to be a very ingenious contrivance as a substitute for a bed; I had a new blanket sewed up in the form of a sack, with a running string at its mouth; into this I got at night, and, tying it round my neck, slept very comfortably on a piece of water-proof sail cloth. The tents under which we lay were not of British manufacture, but a very thin flimsy canvass, pervious to every blast. I continued in perfect health until the retreat of the French permitted us to get under cover of some half-burned villages. After some days spent in marching, I got into a house, and fixed my bed in a room with thirteen other officers, where we were perfectly secure from the inclemency of the weather. My birth was considered as particularly enviable, being in a very dry, sheltered corner; I still used my blanket sack, but the violence of the rains prevented the possibility of exposing it to the air. On the third day I was attacked by irregular chills and febrile heat, and before the tenth my life was in imminent danger from a combination of typhus and dysentery, and nothing but immediate removal to Lisbon preserved it. Three persons, who, in succession, used my blanket, and got into a snug corner, were attacked in the same manner, while all those who slept under the window, or in the more exposed parts of the buildings escaped all febrile affection whatever." p. 176—177

The 13th chapter, though of the utmost moment to the hospital surgeon, is not so interesting to the general practitioner. The subject is *hospital gangrene, as it appeared in some of the British hospital establishments in Spain, Portugal and the Netherlands, and of the ordinary mortification of gunshot wounds.* To give any thing like an analysis of hospital gangrene, as Mr. Hennen describes it, would lead us beyond our object. Of the ordinary mortification of gunshot injuries we have nothing more to say than that Dr. Hennen, with that liberality of spirit which characterizes every truly great man, adopts that masterly division of gangrene, which the Baron Larry has instituted into spontaneous and traumatic. This division is fraught with the most important practical results. We dare not amputate in spontaneous mortification while the disease continues to spread. We must wait till the absorbents have commenced their silent labours ; till they have marked out their field of operations by their red line. As to traumatic mortification, the rule which the Baron discovered and which the experience of many of the most distinguished military surgeons has verified, allows that when mortification is the result of a mechanical cause, and puts the patient's life in danger, we may safely amputate, even before the diseased action has ceased its progress. We would anxiously wish to impress this practical precept on the mind of every practitioner.

Tetanus is still one of the approbria chirurgiæ notwithstanding Hennen's fourteenth chapter.

The 15th chapter, which treats on *amputation*, is a long one and as might be anticipated, rich in useful precepts. In that tremendous operation, the lopping away of the thigh at the hip joint, he recommends Dr. Veitch's mode, as being by far the most preferable. We believe that very few of our surgeons have seen the little *brochure* that contains the method. We make no apology for the following extracts.

"The patient is to be placed on a table, of the height usually employed in amputation, when the tourniquet is to be applied in the groin, so as to act on the superior part of the femoral artery. The assistants will now secure the patient, support the limb, and retract the integuments, as in cases of ordinary amputation. This being done, and the surgeon placed on the outside of the thigh, he will commence his first circular incision, on the same principles as in the ordinary amputation of the thigh, and carry it through the integuments and fascia, freely into the substance of the subjacent muscles, which the assistant will immediately retract, to enable the surgeon to commence, without delay, a second circular incision, at a point somewhat higher, to be carried directly to the bone. When the muscles are completely divided, the next object is to cut the bone an inch or two below the circular incision; with this view a longitudinal incision is to be made on the inside, and another on the outside of the thigh, commencing each incision at the top of the inferior section of the muscles, and carrying it towards the knee, somewhat more than two inches from the place of its commencement. This being done, the muscles are to be dissected from the bone, in the direction of the knee, to the extent of an inch and a half, near which point the bone must be divided. This part of the operation, it is evident, can give no pain, and will not occupy half a minute. The next step is to secure all the blood-vesssel, as in ordinary amputation, to which the removal of the leg, in the manner directed, gives the most perfect access. The blood-vessels being all properly secured, the tourniquet must be removed, and the patient is now to be placed on the sound side, when an incision is to be made, commencing about two inches above the trochanter, and continued in a direct line with that projection, to the extremity of the stump. We are now to consider the bone as a tumour or foreign body, to be removed by expert and steady dissection, commencing from below upwards, and detaching, in the direction of the acetabulum, the adhering muscles on each side. The muscles attached to the trochanter being freely cut, the capsular ligament must be divided circularly, from within outwards, or *vice versa*, according to the thigh operated on, (following the margin of the socket) and during this process, an assistant must press the bone towards the opposite thigh, which will force it from its socket, and enable the surgeon to lay hold of the head of it, and easily separate the attachments of the muscles from the inside of the bone, a step, in this operation, which ought always to be deferred until the head of the bone is thoroughly dislodged, by the division of the ligamentum rotundum.

It must now be evident, that the reason of my leaving a part of the thigh bone projecting beyond the muscles is, to enable my assistant, or the surgeon, to use it in some degree as a lever, to accomplish its own removal. From the nature and extent of the parts divided, in the second stage operation, there would be some reason for apprehension, did the surgeon not possess the most perfect access to, as well as command over, every vessel which he divides. Time is no object; the safety and recovery of the patient is the only consideration; the surgeon is, therefore, when he divides a vessel of any consequence, to make his assistant cover it with his finger, or a sponge, and deliberately to take it up.

The ligatures in the course of the longitudinal wound, are to be gently and easily drawn out, laterally, and as nearly as possible on a line with the mouths of the vessels secured, and the wound is to be closed with adhesive slips. The circular wound is next to be closed, when the same principle, as to the distribution of the ligatures, is to be attended to, as recommended in the paper just noticed. The ligatures are to lie easy, attached to the extremities of the divided vessels, and particular care is to be taken in the application of the adhesive slips, to avoid extending them like vibrating cords. This wound is likely to heal with great rapidity, as the removal of the bone takes away the resistance it would give to the union of the soft parts, and which, to a certain degree, it does in every case of ordinary amputation; and, independent of resistance, its hard, sharp edge, and rough extremity, may act on parts so acutely sensible from recent division, to a certain degree, as a foreign body, until the economy of the system adapts itself to the loss which the constitution has sustained. It is almost unnecessary to recommend, in the removal of the thigh bone by dissection, keeping the knife as close to the bone as possible, in the circular dissection, as well as in separating from above downwards, or *vice versa.* The distance at which the incision is to be begun from the superior part of the thigh, must be ascertained by the circumference of the thigh to be operated on, and then measuring the half of its diameter from the pubic side of the thigh. The mode of operating which I have just described, may be, in many cases, usefully extended to amputation at the shoulder joint."

The 16th is a long chapter devoted to *wounds of the head.*—From the frequency with which sabre and gunshot wounds of the head occur in warfare, we ought to expect that military surgery would present a noble contribution to our mass of knowledge of these injuries. A careful perusal of the chapter before us, will show that such expectations are not unfounded. Dr. Hennen, with that spirit of generalization which is the attribute of wisdom and long experience, sweeps away, at one brush, the cobweb distinctions of the schools, which he considers as "excessive refinements," and at once declares it to be the business of the surgeon in these injuries, to prevent, or subdue inflammation.

"To effect this desirable object, nothing should be omitted in serious injuries of the parts, (and who has not seen apparently the most simple, terminate seriously?) to remove every source of irritation.—We now-a-days, it is true, do not cut away the injured scalp, or procure artificial exfoliation of the uncovered bone; but I certainly think we but too often omit making ourselves perfectly acquainted with their state, by being content with a superficial incision, and clipping the hair surrounding an injury, instead of a free opening, and shaving to a sufficient extent, as practised by our forefathers. Independent of the more accurate view we procure by these means, we facilitate the application of leeches, if they may be found necessary, and of a most excellent adjuvant on all occasions, viz., cold applications, which are ever soothing to the patient, and often materially assistant to his recovery. The formula recommended by Schmucker, is nitre sixteen ounces, muriate of ammonia eight ounces, dissolved in forty pounds of cold water, with the addition of eight pounds of vinegar. To avail ourselves of the full frigorific effects of this mixture, it should be prepared in small quantities, and used immediately before its temperature, (which is greatly depressed by the act of solution,) has risen to that of the surrounding atmosphere. Snow, or pounded ice, or ice water, applied to the parts in a half filled bladder, or cloths simply dipped in cold water, will often answer every purpose.

I think also that I have observed a much less frequent use of the very powerful auxiliary of nauseating doses of antimonials than their utility warrants; and, although I would not go so far as Desault and other French surgeons have done in the recommendation of them, I certainly am of opinion that, in the British military hospitals, they have not generally met the attention they are entitled to. By the employment of these external and internal means; by the use of mild saline purgatives, preceded by the common blue pill; by quiet, and by abstinence, we will often prevent altogether those troublesome puffy enlargements and erysipelatous affections of the scalp, which so often succeed to bruises. And I may here observe, that those extensive and formidable erysipelatous affections, so common formerly, are rare and mild at present in military hospitals, where the evacuant plan is duly observed, and cleanliness and ventillation properly attended to; while in the civil establishments, the affections of the skin in acute diseases are also most remarkably diminished." p. 228—229.

Remarkable cases are mentioned by our author, of injuries of the head, of the most violent and complicated character, in which the patients recovered. So many instances of this nature are on record, that we ought always to bear the *nil desperandum* as our motto. The aphorisms of Vic D'Azyr, drawn from the results of an extensive practice, and a still more extended research, ought to be remembered by every surgeon. The first aphorism of this illustrious anatomist, is, that the largest wounds of the head are not always the most dangerous. 2d. That a considerable portion of the brain may be lost, without death being the necessary consequence. 3d. That the slightest injuries of the head sometimes lead to a fatal termination. 4th. That contusions of the bone alone, sometimes extend so as to affect the brain. Fodere Tom. 3. p. 312.

The question as to the use of the trephine in depression, we consider as settled. We think it ought not to be applied, unless symptoms of compression of the brain exist.

We cannot give even an analysis of the valuable matter contained in this chapter. We offer no apology for the length of the following extract:

"Captain B——, a particular friend of mine, was wounded by a musket ball in the head at Waterloo, on the 18th of June, 1815. On the 19th, he was brought into the city of Brussels, in charge of a medical officer, who gave me a most melancholy account of his case. On approaching the wagon in which he was conveyed, I was insensibly attracted to that part of it where he was stretched, by a low protracted moan, as of a person in extreme pain, but very weak. On calling him by name, he sat up, caught me by the hand, which he kissed most fervently, pointed to his head, and then to the site of a former wound which he had received at the storming of Badajoz, in 1812, from the effects of which I had the good fortune to relieve him. He then burst into tears, but without having the power of uttering a distinct word. His countenance was pale and ghastly, and his mouth somewhat distorted; his eye languid, and suffused with blood; his skin dry, but cool; his pulse about 90, soft, and compressible. As I found that he had been bled on the field, I contented myself with providing him a billet, and giving him in charge of his medical attendant, with directions to examine the wound most particularly; to enlarge it if fracture to any extent appeared; to administer a brisk purge, and to watch most carefully the approach of inflammation. The wounded being now pouring in by hundreds, I was unable to see him before the 21st; his case, however, was reported to me daily. Much coagulated blood, and some particles of sand on which he had fallen, together with a thin scale of lead, obviously a bit of a split musket ball, had been removed. His pulse had risen on the night of his arrival to about 100, hard, and bounding, and he had been copiously bled in consequence. A cruciform enlargement of the wound had been made, which bled copiously, and gave a view of an extensive fracture of the left parietal bone. On my visit, I found him nearly as follows:—Countenance pale, expressive of great pain, referrible more to mental than corporeal suffering, mouth still distorted; eye sunk, but its pupil dilatable; the power of articulating any distinct sound lost, but the desire obviously strong; pulse 80, soft; tongue clean, bowels open, (by saline purgatives) urine copious, and with a rose-coloured sediment; skin moderately warm, and at the region of the liver bathed in sweat; the liver itself obviously projecting, and giving a painful sensation when pressed upon, evinced by his wincing from the touch.

On examining the wound of the head, I found an extensive radiated fracture, occupying almost the whole of the left parietal bone; at the centre there was a piece of bone, apparently the size of a musket ball, beat in through the membranes of the brain, and bedded in its substance, but considerably more toward the frontal region than the occipital. The unequal pressure I found to proceed from a musket ball which was wedged in between the displaced pieces of bone and the portion, which, though cracked, preserved its situation. The separated piece was obviously much more extensive on its internal face than externally, and could not possibly be extracted without the operation of trephining, to which I proceeded. The leaden wedge, and several loose splinters which jammed it in, were easily removed; and on making one perforation with a large sized trephine, I removed the depressed portion of bone, which was forced into the brain nearly an inch and a half from the surface of the scalp. It was of an irregularly oval shape, about one inch long by half an inch broad, and fractured in such a manner, that the internal table formed a much larger part of its circumference than the external.

No relief followed the operation; he passed an extremely restless night, and the pulse rose so rapidly and so high, that the abstraction of sixteen ounces of blood became necessary. His breathing during this momentous night became, for the first time, permanently stertorous; and, when I saw him in the morning, his whole appearance indicated the most extreme danger. He lay *coiled up* in the bottom of his bed; the right arm stretched out, and occasionally convulsed; no exertion could get a sight of his eyes, or his tongue; the mouth was more distorted than usual; the skin was nearly as on the day of operation, except that the partial sweating over the hepatic region was increased in profuseness, and he seemed to wince more on pressure at that part; indeed, all the sympathies seemed to be entirely merged in those connecting the brain and liver. The stomach participated remarkably little, for he had scarcely any vomiting. His pulse alone gave me some hopes; it was nearly natural. On addressing him, he made an effort to rouse himself, but almost immediately relapsed into his former state. I directed a strict watch to be kept over him; and as my duties called me again to that part of the city where he was lodged, I visited him about midnight, and found that a spontaneous bilious diarrhœa had come on, and that he was much more sensible. He made an attempt to articulate, and pronounced audibly the letter T once or twice.

The next morning, being the 5th from the receipt of his wound, his general appearance was amazingly altered for the better; the diarrhœa still remained, and his efforts to speak were continual. On the sixth day he grasped my hand with great fervour, looked piteously in my face, and, to my inquiries as to his feelings, he uttered audibly, though with much labour, the monosyllable "THER," to which, in the course of the day, he added, "O;" and for the three next days, whenever addressed, he slowly, distinctly, and in a most pathetic tone, repeated the words, "O; THER: O; THER:" as if to prove his powers of pronunciation. His general appearance, during all this time, amended considerably, and my hopes now began to revive. I therefore resolved to write to his family, and, before doing so, I printed in large characters, on a sheet of paper, the following words,—"SHALL I WRITE TO YOUR MOTHER?" that being the wish which it appeared to me he so long and ardently had laboured to utter. It is impossible to describe the illumination of his countenance on reading these talismanic words; he grasped and pressed my hand with warmth, burst into tears, and gave every demonstration of having obtained the boon which he had endeavoured to solicit.

From this period his mental faculties gradually developed themselves; he regained a consciousness of the circumstances immediately preceding his wound, and, in succession, of those of a more remote period. The power of speech was the last which he perfectly regained, and for which he usually substituted the communication of his thoughts and wishes in writing. Throughout the whole of his convalescent state, melancholy ideas constantly predominated, although, previous to the accident, he had been remarkable for his flow of spirits. He returned to England, nearly recovered, on the 29th of September, or 103d day from the wound. p. 248—249—250"

Chapter 17th, *on injuries of the eye, ear, face, and neck*, is replete with curious and instructive cases and precepts.

The 18th chapter is devoted to *wounds of the thorax*. The treatment of wounds penetrating the chest, is admirably and forcibly depicted in the following words:

"In whatever part of the thorax a ball, bayonet, or sabre strikes, our first object is to diminish the quantity of circulating blood, so vast a proportion of which passes through the contents of the cavity. On this the very existence of our patient depends; and we cannot, from reasoning *a priori*, fix any bounds to the quantity to be taken, or determine the intervals at which it is to flow; our practice in both respects must be governed by the effects. There lies a man with a wound in his chest; the blood is oozing from the external orifice in a constant, though slow, florid track; in his frequent and painful efforts to cough, it is thrown up in frothy arterial mouthfuls, mixed with occasional clots; his breathing is oppressed almost to suffocation; his pulse quick, weak, and fluttering; his eyes are starting from their sockets; his nostrils are distended in his efforts at relief by inspiration; and his extremities are cold, and often tossed about in fruitless anguish. This wretched being must assuredly die if surgical aid is not promptly afforded him. The mode which should instantly be adopted in such a case is as follows: Without searching after balls or fragments of bone, or attempting to ascertain the precise track of the bayonet or the pike, or expatiating (as I have seen done by some young gentlemen fresh from their studies) upon the particular vessels or their branches which may be injured; let the man lay quietly along, and lose from thirty to forty ounces of blood from his arm, by a large orifice. This done, we should remove the cloths or handkerchief which may have been hurriedly put over the wound to staunch the blood. If he has fainted during the bleeding, or if we find him in that state when we arrive, instead of administering any cordials to him, we should put our finger into the wound, and extract every thing within reach, whether cloth, ball, iron, wood, splinters of bone, or clots of blood. If the orifice is not sufficiently large, we must not be afraid of making it moderately larger, by a cautious use of the probe, pointed bistoury, or the sharp one with a small morsel of wax on the end of it: by this means we make way for the removal of extraneous bodies, and may possibly discover the bleeding orifice of one of the intercostal arteries, which sometimes are cut, but *not at all so often* as speculative writers would lead us to believe. We now proceed to dress the wound itself. If it is gunshot, a light mild dressing will be sufficient; but if incised, the lips should be closed at once; and this treatment will be found to afford the most certain preventive to emphysema, future hemorrhage, or collections of matter. I scarcely recollect an instance where it was necessary to remove the adhesive straps, or (where it was gunshot) the usual dressings. We now lay the man down, and let him remain as quiet as possible, and in as cool and airy a spot of the barn, church, or hospital, as we can find. He will often require no farther aid; but if the case is very severe, he will possibly lie for some hours in a state of comparative ease, till the vessels again pour forth their contents, and induce fresh spitting of bloody froth, and a repetition of all the symptoms of approaching suffocation. The lancet must again be had recourse to; and if, by this management, repeated as often as circumstances demand, the patient survives the first twelve hours, hopes may begin to be entertained of his recovering the immediate effects of hemorrhage. In the after treatment of a wound of the nature here described, we shall be considerably assisted by the aid of medicine: but until the danger of immediate death from hemorrhage is over, we must not think of employing any thing except depletion by the lancet; it, and it only, can save the life of the wounded man." p. 296—297—298.

Dr. Hennen speaks of Emphysema. This subject, for the present, we pretermit, intending at a future period to lay some observations on it before the readers of the Western Journal.

The 19th chapter is *on wounds* of the abdomen, pelvis, &c.—This is a very extensive subject, and we have already rather exceeded our limits.

The remaining chapters *on Miscellaneous points connected with Military Surgery*, on *Variola*, on *Vaccination*, and on *Syphilis*, we cannot notice.

We understand an extensive view of the non-mercurial mode of treating Syphilis, is in preparation for this journal, and will be published in an early number.

In concluding, we have to return our thanks, in common with the rest of our profession, to those enterprising booksellers, Carey & Lea, for having presented this valuable treatise to the surgeons of our country. The appearance of the work is neat, and the execution correct. We most heartily commend it to the patronage of our Western practitioners.

